# Editorial for Special Issue “Technological Advances Around Next-Generation Sequencing”

**DOI:** 10.3390/cimb48010083

**Published:** 2026-01-14

**Authors:** Gaurav Tripathi

**Affiliations:** Kashi Clinical Laboratory, Portland, OR 97219, USA; gtripathi@kashilab.com

Over the past three decades, advances in high-throughput technologies have played a major role in the transformation of biomedical science, which has enabled unprecedented exploration of genomes, transcriptomes, and proteomes [1,2,3]. This Special Issue brings together a collection of studies that exemplify the convergence of next-generation sequencing, genome engineering, artificial intelligence (AI), and molecular analysis to address both fundamental biological questions and clinical challenges [4]. Moreover, papers and reviews in this issue underscore how technological progress continues to refine our understanding of genetic complexity, disease mechanisms, and molecular evolution.

Recent Advances in Sequencing and Molecular Technologies

A common theme in all these contributions included in this Special Issue is how next-generation sequencing has changed clinical study and medicine. From personalized oncology to plant genomics, NGS has developed into a key component of contemporary molecular biology, ranging from plant genomics to cancer biology. Recent reviews and publications in oncology demonstrate how precision medicine and the customization of targeted medicines are informed by thorough genetic profiling using NGS [5] for targeted therapies, leading to a better outcome [6]. Clinicians can now find relevant mutations and maximize therapy responses because of the inclusion of bioinformatics tools into NGS procedures [7]. This development is further demonstrated in a clinical instance of non-small-cell lung cancer (NSCLC) with uncommon co-occurring EGFR and KRAS mutations [8] in a publication by Tushir et al. [9] and Valcz et al. [10], highlighting the necessity of ongoing genetic testing to direct adaptive therapy.

Furthermore, technological refinement is equally evident in the field of library-based selection and molecular screening. The application of NGS to peptide phage display published by Sell et al. [8] and Brown KC [11] revealed hidden biases introduced by fast-propagating phage clones, demonstrating how deep sequencing can uncover pitfalls in conventional selection methods and enhance ligand discovery accuracy. Similarly, the recent development of Center Degenerated Walking-Primer PCR, a molecular technique for mining unknown flanking DNAs, published by Tang et al. [12] and Gao et al. [13], introduces a robust, universal genome-walking method with improved specificity, expanding the toolkit for genomic exploration.

On the other hand, a paper published by Chellappan et al. on ‘Assembly and Comparative Analysis of Complete Mitochondrial Genome Sequence of Endangered Medicinal Plant Trichopus zeylanicus’ [14] provides an essential genomic resource for understanding plant evolution and conservation biology. This effort, combining Illumina and PacBio sequencing which creates powerful hybrid genome assembly strategies, underscores the growing impact of hybrid approaches in resolving complex genomic architectures [15].

Integrating Artificial Intelligence and Advanced Analytics

In this Special Issue, we have one of the most advanced tool integrated with genomics; this is artificial intelligence (AI), often called ‘GenomicAI’, where the combination of AI and genetics has become a key area of research [16]. As discussed by Athanasopoulou et al. [17], the incorporation of machine learning and deep learning models into NGS analysis pipelines has accelerated variant detection, transcriptomic profiling, and epigenomic interpretation [18]. These intelligent frameworks not only enhance accuracy but also facilitate automation and scalability in both research and clinical diagnostics. Importantly, the review highlights future directions including federated learning, interpretable AI, and multi-omics data integration, which will be vital for ethically robust and clinically trustworthy implementation [17].

Pattabiram et al. [19] defined in parallel, single cell sequencing and spatial transcriptomics which revolutionizing our understanding of the tumor microenvironment leading to better diagnostics and personalized therapies. These technologies make it possible to see how genes work in individual cells and how these cells interact with each other. This helps us understand how cancer growth and treatment resistance are affected by the complexity of these relationships and the differences between the cells [20]. Together, these approaches mark a significant move toward more precise, systems-level biology.

Expanding Beyond the Genome: Proteomics and Functional Complexity

The study of the dark proteome in *Solanum lycopersicum* published by Reyes-Soria et al. [21] reminds us that much remains unknown beyond the sequence level. By describing the structural order and fundamental disorder in proteins that have not been annotated, we can find new ways to explore functional “blind spots” in proteomics. This could have an impact on how we respond to stress and improve crops. Another study published by Urban et al. [22] in this Special Issue on CRISPR-Cas9 technology [22] where he continues to transform both basic and applied biology, from engineering animal models to improving livestock genetics and producing therapeutic biomolecules. Menchaca et al. [23] discussed how CRISPR can be used to improve livestock production to meet the growing global demand for food. They highlighted its potential to increase efficiency, reduce environmental impact, improve pest control, and support better animal health and welfare. These examples show the amazing capabilities of precise genome manipulation.

Knowledge Gaps and Challenges

Despite the remarkable advances showcased in this Special Issue, several knowledge gaps persist and there is always a scope of improvement; however, together with the AI and integrative genomics, the following suggestions can be implemented:

- Standardization and data interpretation: These issues continue to be major challenges in NGS and AI-driven research. Interoperable frameworks and reliable validation procedures are necessary due to the sheer volume and diversity of multi-omics data.
- Proteome and plant mitochondrial genome investigations: These studies show that we are discovering new sequences faster than we can understand what they do. A major challenge is still connecting genes to their actual effects in living organisms.
- Tumor heterogeneity and therapy resistance: These issues continue to make precision cancer treatment difficult. They require models that combine information about where tumors are, how they change over time, and their molecular details.
- Ethical and regulatory considerations: To keep public trust, we need clear and collaborative guidelines, especially for AI, gene editing, and patient data. This Special Issue aims to address these challenges through interdisciplinary work that advances sequencing accuracy, molecular diagnostics, and integrated bioinformatics.
- Insurance coverage and clinical validity: A significant proportion of identified variants are classified as variants of uncertain significance (VUS), particularly in underrepresented populations. The presence of VUS limits clinical actionability and contributes to hesitation among clinicians, payers, and researchers [24,25,26]. Insurance coverage often depends on clear clinical validity and utility, which VUS by definition do not yet provide.

Future Directions

Future directions in the subject include multi-omics and AI-assisted precision biology. Integrating genomic, transcriptomic, proteomic, and spatial data within unified computational frameworks will enable our deeper understanding of disease mechanisms and associations and evolutionary biology. The future research should focus on the development of interpretable AI methods, the expansion of real-time sequencing in clinical and field settings, improving functional annotation of genomic and proteomic data that are not well characterized, and the promotion of sustainable and ethical genome editing practices for biomedical and agricultural applications. However, robust genotype–phenotype correlations, supported by large datasets and longitudinal clinical evidence, are required before widespread adoption can be achieved since these tools remain complementary and investigational.
